# Association of interleukin 22 receptor subunit alpha 1 gene polymorphisms with chronic rhinosinusitis

**DOI:** 10.1016/j.bjorl.2019.10.006

**Published:** 2019-11-15

**Authors:** Vanessa R. Pires Dinarte, Wilson A. Silva, Anemari R.D. Baccarin, Edwin Tamashiro, Fabiana C. Valera, Wilma T. Anselmo-Lima

**Affiliations:** a Faculdade de Medicina de Marília, Divisão de Otorrinolaringologia, Marília, SP, Brazil; b Universidade de São Paulo, Hospital de Clínicas da Faculdade de Medicina de Ribeirão Preto, Departamento de Genética e Centro de Genômica Médica, Ribeirão Preto, SP, Brazil; c Instituto Nacional de Ciência e Tecnologia em Terapia Celular e Células-Tronco, Laboratório de Genética Molecular, Centro de Terapia Celular, São Paulo, SP, Brazil; d Universidade de São Paulo, Faculdade de Medicina de Ribeirão Preto, Divisão de Otorrinolaringologia, Ribeirão Preto, SP, Brazil

**Keywords:** Nasal polyps, Chronic rhinosinusitis, Polymorphisms

## Abstract

**Introduction:**

Chronic rhinosinusitis is a multifactorial disease whose pathogenesis, influenced by both genetic and environmental factors, is still unclear. Previous genetic studies have shown that patients with chronic rhinosinusitis have reduced expression of the Interleukin-22 (IL-22) gene.

**Objective:**

Identify and compare the frequency of polymorphisms in the IL22RA1 gene (IL22 alpha-1 subunit receptor) among chronic rhinosinusitis patients – either with or without nasal polyps.

**Methods:**

Peripheral blood samples were collected from 70 chronic rhinosinusitis with polyps patients, 14 chronic rhinosinusitis without polyps patients and 68 subjects without chronic rhinosinusitis, followed by DNA extraction and IL22RA1 gene sequence analysis.

**Results:**

Among ten polymorphisms identified in the IL22RA1 gene, three were not found in any of the genetic databases analyzed. Chronic rhinosinusitis patients displayed higher frequency of the c.113_114insA frameshift insertion, possibly pathogenic. Conversely, in the control group, polymorphism c.435A > C had a significant predominance of the mutated allele, perhaps related to a potential protection against the chronic rhinosinusitis phenotype. Polymorphism c.770C > T, characterized as a non-synonymous variant, was exclusively found in Black chronic rhinosinusitis with polyps patients.

**Conclusions:**

Although no direct causal relationship could be established between IL22RA1 gene polymorphisms and the pathophysiology of chronic rhinosinusitis, genetic variations such as c.113_114insA and c.435A > C may be involved in the susceptibility to or protection against the chronic rhinosinusitis phenotype, respectively. Testing this hypothesis will require studies with larger cohorts.

## Introduction

Despite the identification of several factors as potentially involved in chronic rhinosinusitis (CRS) – including allergic responses, impaired mucociliary clearance, immune dysfunction, altered epithelial defense, microbial agents, and environmental exposure^1^ – CRS etiology remains unclear. A more assertive management of the patients requires further clarification on how these factors interact with each other, as well as with genetic factors, in the etiology of CRS.

IL-22 is a cytokine of the IL-10 family that may be involved in acute phase inflammatory response, innate immunity system activation, induced cell migration, dendritic cell inhibition, and attenuation of allergic responses.^1^, ^2^ The IL22RA1 receptor may also play an essential role in tissue healing and reorganization. Kotenko et al.^3^ showed that IL22RA1 levels were significantly lower in the epithelial cells of CRS patients with NPs (CRSwNP) who did not respond to surgery. This could result in damage to the epithelial barrier, with a decrease in the production of Th1 pro-inflammatory cytokines, causing an inversion in the Th1/Th2 response pattern. Alternatively, changes in the heterodimer regulatory complex (IL22RA1-IL10R2, IL20, and IL24) could lead to decrease in the innate immune response (β-defensins, mucins and metalloproteinases), increased levels of pro-inflammatory cytokines (IL-6, IL-8, and TNF), and reduced apoptosis,^3^, ^4^ providing other possible pathophysiological mechanisms involved in the development of CRS.

Although genetic variants might not be the origin of a given disease, they may confer susceptibility to its development. Therefore, the present study aimed to identify variants associated with the IL22RA1 gene in Brazilian patients diagnosed with chronic rhinosinusitis and in individuals without this diagnosis.

## Methods

The choice of the IL22RA1 gene resulted from a previous Canadian DNA clustering survey (pool-based Genome-Wide Association Study)^5^ that selected five high-priority Single Nucleotide Polymorphisms (SNPs) and another 18 polymorphisms from the HapMap international project.^6^ Amongst these 23 SNPs, the IL22RA1 gene was a strong candidate for the development of CRS.^5^

The present study was approved by the Research Ethics Committee of the Clinical Hospital of the Ribeirão Preto Medical School of the University of São Paulo (HCFMRP-USP) and the National Commission for Research Ethics (CONEP), under Protocol nº 5243/2011. All patients and control subjects were invited to participate in the survey after clarification of study procedures and objectives, sample collection method, risks, and possible access to the results, as indicated in a Free and Informed Consent Form (ICF).

Patients and control subjects were recruited at the Otorhinolaryngology Outpatient Clinic of the HCFMRP-USP. According to the clinical criteria of the 2012 European position paper on rhinosinusitis and nasal polyps guideline (EPOS),^7^ patients were classified as having CRS with or without NPs. The control group comprised 68 volunteers with no nasal symptoms and with normal nasofibroscopy, unrelated to the participants in the other two groups. All participants filled out a questionnaire with their name, age, gender, current and remote origin, tobacco use, skin color and current disease history, including information on seasonal and perennial allergies, asthma and intolerance to acetylsalicylic acid. Patients with Churg-Strauss syndrome, primary ciliary dyskinesia, and cystic fibrosis were excluded from the study.

Plasma was extracted from the peripheral blood samples by centrifugation at 2500 RPM for 10 min at 4 °C. Afterwards, DNA was extracted from the “buffy coat”. The seven exons of the IL22RA1 gene were amplified by Polymerase Chain Reaction (PCR), with the seventh exon divided into five sequences, due to its size, to simplify the analysis. The PCR protocol included denaturation at 95 °C, followed by annealing, at temperatures chosen according to the primer used and DNA extension at 72 °C, repeated for approximately 35 cycles until the plateau of the reaction (final phase) was reached. PCR products were analyzed in 1.5 % agarose gels to confirm the amplification and expected size of the fragment.

Each exon was sequenced in the 3500 XL Genetic Analyzer automated sequencer (Applied Biosystems), using the Sanger method. The sequencer output file consists of a chromatogram that contains the information of the sequence and quality of the nucleotides.

The pathogenicity of variants was investigated by UMD-predictor,^8^ which combines biochemical properties of mutated amino acids, impact on splicing signals, location of mutations in functional domains, variant frequency in the global population, conservation of mutated amino acids with the global BLOSUM62 substitution matrix, and conservation of amino acid sequences in 100 species. The predictors PolyPhen2^9^ and Sift/PROVEAN^10^ were used to aid the results by the UMD-predictor. Variants were also analyzed with the Combined Annotation Dependent Depletion (CADD) algorithm, which evaluates the degree of mutation pathogenicity, including small insertions and deletions (InDels).^11^ The VarSome platform was used to consolidate all information regarding pathogenicity prediction.^12^

Fisher’s exact test was used to assess possible associations between the analyzed variables. The polymorphisms analysis was performed in different steps: CRSwNP group versus control group, CRSsNP group versus control group, and CRSwNP group versus CRSsNP group (Table 1, Table 2, Table 3).

**Table 1:** Statistical analysis comparing the allelic frequencies in patients with chronic rhinosinusitis with nasal polyps and control subjects.

Nucleotide	Genotype	CRSwNP, n (%)	Control, n (%)	*p*-value
		70	68	
c.135 G > A	G/G	65 (92.9)	59 (86.8)	0.37
	G/A	5 (7.1)	9 (13.2)	
c.135 G > A	G/G	66 (94.3)	68 (100.0)	0.14
	A/A	4 (5.7)	0 (0.0)	
c.113_114insA	WT	57 (81.4)	57 (83.8)	0.88
	Ins A	13 (18.6)	11 (16.2)	
c.74 T > A	T/T	70 (100.0)	67 (98.5)	0.99
	A/A	0 (0.0)	1 (1.5)	
c.141C > A	C/C	70 (100.0)	67 (98.5)	0.99
	C/A	0 (0.0)	1 (1.5)	
c.435A > C	A/A	50 (71.4)	33 (48.5)	**0.01**
	A/C	20 (28.6)	35 (51.5)	
c.435A > C	A/A	37 (52.9)	47 (69.1)	0.07
	C/C	33 (47.1)	21 (30.9)	
c.613 G > A	G/G	70 (100)	68 (100)	N/A
	A/A	0	0	
c.770C > T	C/C	68 (97.1)	68 (100.0)	0.49
	C/T	2 (2.9)	0 (0.0)	
c.936C > T	C/C	67 (95.7)	64 (94.1)	0.97
	C/T	3 (4.3)	4 (5.9)	
c.1077C > T	C/C	69 (98.6)	68 (100.0)	1.00
	C/T	1 (1.4)	0 (0.0)	
c.1552C > G	G/G	67 (95.7)	65 (95.6)	1.00
	C/C	3 (4.3)	3 (4.4)	
c.1552C > G	G/G	70 (100.0)	67 (98.5)	0.99
	G/C	0 (0.0)	1 (1.5)	

CRSwNP, Chronic Rhinosinusitis with Nasal Polyps, N, Number of samples, C, Cytosine, T, Thymine, A, Adenine, G, Guanine, WT, Wild Type, N/A, Not Applicable.

p<0,01: possible protection against the phenotype

**Table 2:** Statistical analysis comparing the allelic frequencies in patients with chronic rhinosinusitis without nasal polyps and control subjects.

		CRSsNP, n (%)	Control, n (%)	*p*-value
		14	68	
c.135 G > A	G/G	13 (92.9)	59 (86.8)	0.85
	G/A	1 (7.1)	9 (13.2)	
c.135 G > A	G/G	14 (100.0)	68 (100.0)	N/A
	A/A	0	0	
c.113_114insA	WT	13 (92.9)	57 (83.8)	0.65
	Ins A	1 (7.1)	11 (16.2)	
c.74 T > A	T/T	14 (100.0)	67 (98.5)	1.00
	A/A	0 (0.0)	1 (1.5)	
c.141C > A	C/C	14 (100.0)	67 (98.5)	1.00
	C/A	0 (0.0)	1 (1.5)	
c.435A > C	A/A	9 (64.3)	33 (48.5)	0.44
	A/C	5 (35.7)	35 (51.5)	
c.435A > C	A/A	8 (57.1)	47 (69.1)	0.58
	C/C	6 (42.9)	21 (30.9)	
c.613 G > A	G/G	13 (92.9)	68 (100.0)	0.38
	A/A	1 (7.1)	0 (0.0)	
c.770C > T	C/C	14 (100.0)	68 (100.0)	N/A
	C/T	0	0	
c.936C > T	C/C	14 (100.0)	64 (94.1)	0.80
	C/T	0 (0.0)	4 (5.9)	
c.1077C > T	C/C	14 (100.0)	68 (100.0)	N/A
	C/T	0	0	
c.1552C > G	G/G	14 (100.0)	65 (95.6)	0.98
	C/C	0 (0.0)	3 (4.4)	
c.1552C > G	G/G	14 (100.0)	67 (98.5)	1.00
	G/C	0 (0.0)	1 (1.5)	

CRSsNP, Chronic Rhinosinusitis without Nasal Polyps; n, number of samples; C, Cytosine; T, Thymine; A, Adenine; G, Guanine; WT, Wild Type; N/A, Not Applicable.

**Table 3:** Statistical analysis comparing the allelic frequencies in patients with chronic rhinosinusitis with and without nasal polyps.

	Genotype	CRSwNP, n (%)	CRSsNP, n (%)	*p*-value
		70	14	
c.135 G > A	G/G	65 (92.9)	13 (92.9)	1.00
	G/A	5 (7.1)	1 (7.1)	
c.135 G > A	G/G	66 (94.3)	14 (100.0)	0.82
	A/A	4 (5.7)	0 (0.0)	
c.113_114insA	WT	57 (81.4)	13 (92.9)	0.51
	Ins A	13 (18.6)	1 (7.1)	
c.74 T > A	T/T	70 (100.0)	14 (100.0)	N/A
	A/A	0	0	
c.141C > A	C/C	70	14	N/A
	C/A	0	0	
c.435A > C	A/A	50 (71.4)	9 (64.3)	0.83
	A/C	20 (28.6)	5 (35.7)	
c.435A > C	A/A	37 (52.9)	8 (57.1)	1.00
	C/C	33 (47.1)	6 (42.9)	
c.613 G > A	G/G	70 (100.0)	13 (92.9)	0.37
	A/A	0 (0.0)	1 (7.1)	
c.770C > T	C/C	68 (97.1)	14 (100.0)	1.00
	C/T	2 (2.9)	0 (0.0)	
c.936C > T	C/C	67 (95.7)	14 (100.0)	1.00
	C/T	3 (4.3)	0 (0.0)	
c.1077C > T	C/C	69 (98.6)	14 (100.0)	1.00
	C/T	1 (1.4)	0 (0.0)	
c.1552C > G	G/G	67 (95.7)	14 (100.0)	1.00
	C/C	3 (4.3)	0 (0.0)	
c.1552C > G	G/G	70 (100.0)	14 (100.0)	N/A
	G/C	0	0	

CRSwNP, Chronic Rhinosinusitis with Nasal Polyps; CRSsNP, Chronic Rhinosinusitis without Nasal Polyps; n, Number of samples; C, Cytosine; T, Thymine; A, Adenine; G, Guanine; WT, Wild Type; N/A, Not Applicable.

## Results

Overall, 152 samples were analyzed: 70 from CRSwNP, 14 from CRSsNP, and 68 from control subjects. Women were 55 % of the study participants, and their mean age was 46.9 years. Regarding skin color, 80 % stated being White, 9 % Brown/Mulatto 16 %, Black 3 %, and 1 % Yellow. Among CRS patients, 13 % had asthma, with most of these having NPs (90 %). Tobacco use was reported by 3 % of participants, mostly in the CRSsNP group. Of the 19 % of participants who had positive allergy skin tests (Prick test), 86 % had NPs. Of the 9 % of participants intolerant to Aspirin, 92 % were CRSwNP patients (Table 4).

**Table 4:** Characteristics of the study population.

	CRSwNP	CRSsNP	Control	Total
Individuals	70	14	68	152
Female	28 (40%)	10 (71%)	45 (66%)	83 (55%)
Male	42 (60%)	04 (29%)	23 (34%)	69 (45%)
Mean age	50.2	55.9	41.6	46.9
White	59 (84%)	12 (86%)	51 (75%)	122 (80%)
Brown/Mulatto	07 (10%)	2 (14%)	15 (22%)	24 (16%)
Black	04 (6%)	0	01 (1.5%)	05 (3%)
Yellow	0	0	01 (1.5%)	1 (1%)
Asthma	18 (26%)	02 (14%)	0	20 (13%)
Tobacco use	02 (3%)	03 (21%)	0	05 (3%)
Prick test +	25 (36%)	04 (29%)	N/A	29 (19%)
Intolerance to aspirin	12 (17%)	01 (7%)	0	13 (9%)

CRSwNP, Chronic Rhinosinusitis with Nasal Polyps; CRSsNP, Chronic Rhinosinusitis without Nasal Polyps; N/A, Not Applicable.

The sequencing procedure identified 10 polymorphisms in the IL22RA1 gene, located on chromosome 1 (Table 5). Information on the pathogenicity of the polymorphisms c.135 G > A, c.74 T > A, c.141C > A, c.435A > C, c.770C > T, c.936C > T, and c.1077C > T was not available, but c.613 G > A and c.1552C > G have been described as non-pathogenic. The variant c.113_114insA (Gln26Profs*11) is probably pathogenic, a typical characteristic of frameshift mutations (Table 6).

**Table 5:** Description of the polymorphisms found in the IL22RA1 gene.

Exon	Nucleotide	Amino acid	SNP ID	ACMG classification	Type	MAF
2	c.135 G > A	p.Pro45=	rs10903022	Benign	Synonymous	0.47
2	c.113_114insA	p.Gln26Profs*11	ND	2	Frameshift	N/A
2	c.74 T > A	p.Leu25His	ND	Benign	Non-synonymous	N/A
2	c.141C > A	p.Gly47=	ND	Benign	Synonymous	N/A
4	c.435A > C	p.Pro145=	rs17852649	Benign	Synonymous	0.48
5	c.613 G > A	p.Val205ILe	rs16829204	Benign	Non-synonymous	0.15
6	c.770C > T^1^	p.Pro257Leu	rs142356961	Uncertain significance	Non-synonymous	<0.01
7	c.936C > T	p.Pro312=	rs17852648	Benign	Synonymous	0.21
7	c.1077C > T	p.Val359=	rs34967816	Benign	Synonymous	0.12
7	c.1552C > G^a^	p.Arg518Gly	rs3795299	Uncertain Significance	Non-synonymous	0.47

SNP ID, Single Nucleotide Polymorphism Identification; MAF, Minor Allele Frequency; N/A, Not Applicable; ND, Not Described.

Combined Annotation Dependent Depletion (CADD) method generates predictive scores for single nucleotide variants in all areas of the genome, including non-coding regions.

a CADD score over 18, compatible with pathogenic variants.

**Table 6:** Characterization of the polymorphisms found in the IL22RA1 gene in the different study groups.

Nucleotide	Citation	Impact	CRSwNP (70)		CRSsNP (14)		Control (68)	
			Ht	Ho	Ht	Ho	Ht	Ho
p.Pro45=	Yes	LPI	5	4	1		9	
p.Gln26Profs*11	No	PATH?	13		1		11	
p.Leu25His	No	LPI						1
p.Gly47=	No	LPI					1	
p.Pro145=	Yes	LPI	20	33	5	6	35	21
p.Val205ILe	Yes	Non-PATH				1		
p.Pro257Leu	Yes	LPI	2					
p.Pro312=	Yes	LPI	3				4	
p.Val359=	Yes	LPI	1					
p.Arg518Gly	Yes	Non-PATH		3			1	3

CRSwNP, Chronic Rhinosinusitis with Nasal Polyps; CRSsNP, Chronic Rhinosinusitis without Nasal Polyps; LPI, Lacking Pathogenicity Information; PATH?, Probably Pathogenic; NON-PATH, Non-Pathogenic.

Variant c.113_114insA (Gln26Profs*11), which had not been previously described, was found mainly in the CRSwNP. This frameshift variant could result in a nonfunctional protein, possibly contributing to CRS development.

Regarding exon 4 in heterozygosity, only variant c.435A > C (p.Pro145=) presented a statistically significant predominance of the mutated allele in the control group (51.5 %) compared to the CRSwNP group, suggesting a possible protection against the CRS phenotype. However, information on the pathogenicity of this polymorphism is not available in the prediction databases.

## Discussion

In an attempt to identify possible genetic mechanisms that trigger chronic rhinosinusitis, we compared the polymorphisms of the IL22RA1 gene in Brazilian CRS samples and in a control group, without CRS. We identified a novel mutation in the IL22RA1 gene, the c.113_114insA (Q26Pfs*11) frameshift, a potentially pathogenic mutation that appeared primarily in the patient groups, but there was no statistical significance when compared to the control group. Additionally, polymorphism c.435A > C, in heterozygosity, occurred more frequently in the control group than in the CRSwNP group, suggesting that this variant could confer protection against the development of CRS.

Three polymorphisms have never been described in the genetic databases analyzed (dbSNP, ExAC): Gln26PProfs*11, p.Leu25His, and p.Gly47 = . Variant p.Pro257Leu, detected in two Black patients with NPs, retained a Minor Allele Frequency (MAF) of less than 0.01. As this is a variant characterized as of uncertain significance and has a low frequency in the general population, it would be interesting to perform functional assays to confirm its pathogenicity.

The study conducted by Ramanathan et al.^13^ was the first to demonstrate decreased IL22RA1 gene expression in subjects with CRSwNP compared to subjects without polyps and control group, but no explanation was proposed for this difference. A study with Canadian patients, proposed polymorphisms of the IL22RA1 gene as a potential cause for CRS.^9^ However, the IL22RA1 polymorphisms described here are distinct from those described by Mfuna-Endam et al. (2009).^5^ This might be due to the distinct populations from which they were sampled, the different environmental factors to which they were exposed, or even to the smaller number of patients analyzed in our study.

We did not investigate how the mutations observed here could contribute to CRS development, with or without NPs. Patients with CRS are known to have lower IL-22 expression, potentially causing epithelial barrier dysfunction and changes in Th1 response, and the inflammatory imbalance between Th1 and Th2 could contribute to CRS development. Variations in the IL22RA1 gene might dysregulate the innate immunity mechanisms, leading to decreases in specific inflammatory mediators. Impairment of the heterodimer complex comprising IL-10R2, IL-20 and IL-24 is another mechanism that could hamper signal transduction and transcription activation (JAK/STAT pathway), leading to dysregulation of pro-inflammatory cytokine levels.

Despite resulting in alterations that may lead to changes in protein configuration, the incidence of the detected polymorphisms was low. Further studies are required, within larger cohorts, to decrease the likelihood that these findings were random, assuring their replicability, and to define the impact of these polymorphisms in the disease in question.

## Conclusions

In this study, we identified a novel polymorphism positively associated with CRSwNP, variant c.113_114insA (Q26Pfs*11). Also, a polymorphism in exon 4 was significantly more frequent in the control group than in the CRSwNP group (c.435A > C in heterozygosity), suggesting a potential protection against the CRS phenotype.

## Conflicts of interest

The authors declare no conflicts of interest.
